# Prospective evaluation of pregnancy outcome in an Italian woman with late-onset combined homocystinuria and methylmalonic aciduria

**DOI:** 10.1186/s12884-019-2474-5

**Published:** 2019-08-30

**Authors:** Elvira Grandone, Pasquale Martinelli, Michela Villani, Gennaro Vecchione, Lucia Fischetti, Angelica Leccese, Rosa Santacroce, Gaetano Corso, Maurizio Margaglione

**Affiliations:** 1 Fondazione IRCCS ‘Casa Sollievo della Sofferenza’, Unità di Aterosclerosi e Trombosi, 71013, San Giovanni Rotondo (Foggia), Foggia, Italy; 2 Ob/Gyn Department, The First I.M. Sechenov Moscow State Medical University, Moscow, Russia; 3 Department of Neuroscience, Reproductive Sciences and Dentistry, School of Medicine, Naples, Italy; 4 Dipartimento di Medicina Clinica e Sperimentale, Università degli Studi di Foggia, Foggia, Italy

**Keywords:** Pregnancy, Homocysteine, Metabolic disorders

## Abstract

**Background:**

Cobalamin metabolism disorders are rare, inherited diseases which cause megaloblastic anaemia and other clinical manifestations. Early diagnosis of these conditions is essential, in order to allow appropriate treatment as early as possible.

**Case presentation:**

Here we report the case of a patient who was apparently healthy until the age of 20, when she presented with impaired renal function and normocytic anaemia. At the age of 34, when her first pregnancy resulted in an intrauterine death of a morphologically normal growth-restricted foetus, she was diagnosed with homocystinuria and methylmalonic aciduria due to cyanocobalamin C (cblC) defect, which was confirmed by molecular investigation. Consequently, hydroxocobalamin was administered to correct homocysteine plasma levels. This treatment was efficacious in lowering homocysteine plasma levels and restored anaemia and renal function. During a second pregnancy, the patient was also administered a prophylactic dose of low molecular -weight heparin. The pregnancy concluded with a full-term delivery of a healthy male.

**Conclusions:**

This case emphasises the importance of awareness and appropriate management of rare metabolic diseases during pregnancy. We suggest that women with late-onset cblC defect can have a positive pregnancy outcome if this metabolic disease is treated adequately.

## Background

Cobalamin C (cblC, OMIM #277400) defects are inherited autosomal recessive disorders of vitamin B12 metabolism, with an unknown true prevalence. The defect is an inborn error of intracellular cobalamin (cbl) metabolism due to mutations in the MMACHC gene. The altered activity of this enzyme causes increased levels of methylmalonic acid (MMA) and homocysteine, with reduced levels of methionine. Indeed, methylcobalamin and adenosylcobalamin are not synthesised because of this enzymatic deficiency: the former plays a central role as cofactor of methionine synthase, the latter is a cofactor for the mitochondrial enzyme methylmalonyl-CoA mutase, involved in the degradation of methylmalonic acid [1].

An incidence of cblC ranging from of 1:100,000 to 1:200,000 births has been estimated, with a higher frequency in the Hispanic population (about 1:37,000) [2]. Clinical manifestations and the age of onset of clinical signs and symptoms are variable. About 90% of described patients show the severe, early-onset disease [1, 3].

Late-onset cblC disease is less common than the early-onset one and is usually characterised by neurological complications and anaemia. However, it often has a less severe presentation with a more favourable outcome when treated.

In absence of neurological disease, clinical suspicion and diagnosis may be delayed or overlooked [4, 5].

In 2006 Lerner-Ellis and coll. first mapped the cblC locus and identified mutations in the MethylMalonic Aciduria (Cobalamin deficiency) cblC type, with Homocystinuria (MMACHC gene, OMIM*609831) [6].

There is no evidence that females have a milder course of the disease. A male/female ratio of about 2 in early- onset disease has been calculated. Interestingly, at variance with the early- onset, the late-onset disease is more frequent in females, with a male/female ratio of 0.7 [5].

Here, we present two pregnancies in a woman whose first pregnancy ended with the occurrence of an apparently unexplained Intra-Uterine Foetal Death (IUFD). Consequently, she was diagnosed with late-onset cblC disease, metabolic indexes were corrected and the woman initiated a second pregnancy which was successfully brought to full term.

## Case presentation

A 34-year-old Caucasian woman from Southern Italy came to our attention because of a late (20th week gestational age) pregnancy loss of a morphologically normal intrauterine growth restricted foetus. The placenta, weighing 80 g, showed severe maturation alterations, intervillous thrombosis, fibrin deposition, especially on the maternal side and multiple areas of infarction. Pathologists concluded with “severe vascularization abnormalities, similar to those observed in severe preeclampsia”. Therefore, the patient underwent a thrombophilia screening, that showed 100 μmol/L of plasma total homocysteine (tHcy) and she was referred to our Thrombosis and Hemostasis Unit. At that time, she was being administered folic acid (calcium folinate, 15 mg/day) and her folate levels were 11.4 nmol/L (range: 3.1–17.5); B12 serum levels were 722 pmol/L (range: 191–663).

An accurate clinical history was collected together with laboratory data. She had been delivered at full term and had remained in apparently good health until the age of 20, when she showed elevated inflammatory markers and an impaired renal function. The urinalysis carried out revealed proteinuria and microhaematuria. A neurological examination showed normal results. She also presented with a normocytic anaemia (Hb: 8.2 g/L). A blood smear and a bone marrow aspiration were carried out but resulted negative.

A renal biopsy revealed thrombotic microangiopathy (TMA) with predominant lesions in the glomerulus and minimal lesions in the arterioles. However, these findings were not confirmed in another Centre two years later.

These additional data induced physicians to suspect a disease in the metabolic pathway of homocysteine-methionine. Methylmalonic acid was measured and found higher than normal (1.09; μmol/L, reference value: 0–0.7 μmol/L).

After DNA extraction according to local protocol [7], a Whole Exome Sequencing Analysis (WES) was performed. This showed the presence of a compound heterozygosis for p. Tyr130His and p.Tyr222Stop in the MMACHC gene (Methylmalonic Aciduria type C and Homocystinuria) (OMIM * 609831). Both of these mutations have been described by Lerner-Ellis [6].

**Fig. 1:**
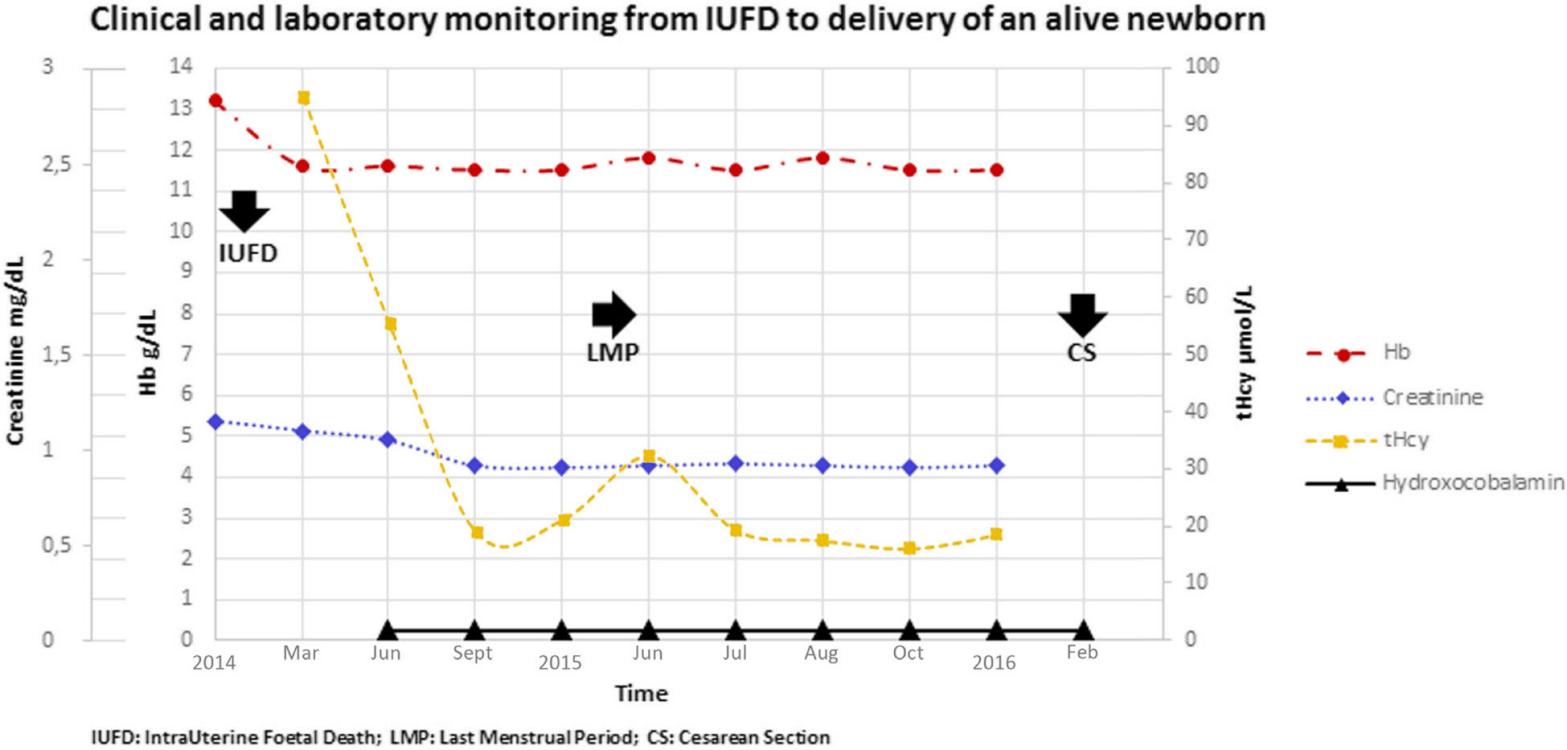
The figure depicts clinical and laboratory monitoring from IUFD to live birth

Thus, in addition to calcium folinate, the patient was prescribed intramuscular hydroxocobalamin, 1 mg/week. This treatment only partially reduced tHcy. Consequently,1 mg every 3 days was used and this approach normalised tHcy plasma levels and restored anaemia and renal function. Once normal levels of tHcy were reached, the patient initiated a second pregnancy. After a positive pregnancy test, she was prescribed low-molecular weight heparin (enoxaparin) at prophylactic doses (4000 IU/day) in addition to hydroxocobalamin. During the pregnancy there were no signs of anaemia or abnormal renal function and at 39 weeks a 2420 g male newborn (Apgar 1′: 8, 10′: 9) was delivered. In the figure (Fig. 1), clinical and laboratory features, from the IUFD to the live birth of the second pregnancy are summarised.

## Discussion and conclusions

To the best of our knowledge, this is the fifth pregnancy described in a patient with Cobalamin C disorder. None of the women with at term pregnancy carried these mutations and all of them were at their first pregnancy. All but one were asymptomatic; indeed, the only woman with previous diagnosis of the disease was identified because of neurological signs at 14 yrs. [8] Most of these inborn disorders are reported in the newborn and infant period, but the wide clinical variability often makes the diagnosis difficult. It has been calculated that about 90% of the patients suffer from the more severe early-onset disease [2, 3]. This form is characterised by feeding difficulties, failure to thrive, psychiatric and neurological disorders. On the other hand, late-onset cblC defects are uncommon and cases so far described in literature were characterised by cognitive and psychiatric problems. As in the case of the patient here described, the diagnosis of late forms of the disease is often overlooked due to its rarity and the absence of extra-neurologic signs [2, 9].

Parenteral hydroxocobalamin is the mainstay of therapy and should be instituted as soon as a disorder of intracellular cobalamin metabolism is suspected. Patients with cblC are often highly responsive to this therapy and usually infants start with daily 1.0 mg intramuscular or subcutaneous injections [10]. We were unsure about whether the correction of metabolic and clinical features would be enough to assure an uneventful pregnancy. Following analysis of data referring to severe alteration of the utero-placental flow and the histology of the placenta, prophylactic doses of enoxaparin were administered.

Most late-onset cblC patients have normal/borderline low haemoglobin with normal B12 plasma levels. Indeed, our patient showed a normochromic normocytic anaemia, that was fully and stably corrected by the administration of hydroxocobalamin. It is worth noting that no signs of anaemia were observed throughout the pregnancy, thus demonstrating that vitamin B12 supplementation was sufficient to avoid anaemia also during pregnancy.

The most recent data show 61 patients suffering from late-onset cblC [5]. 17 of them showed symptoms starting from 18 years old and none of the 10 women described reported a pregnancy. It is conceivable that fertility is not affected in women with late-onset disease but, as demonstrated by the obstetric history of our patient, pregnancy loss can be observed in absence of adequate therapy.

In other metabolic diseases such as phenylketonuria and possibly hyperhomocystinemia, an increased risk of miscarriage has been shown especially in the presence of poor maternal metabolic control.

Thus, it is crucial to discuss with women in reproductive age the value of good metabolic control before conceiving and during pregnancy.

Usually diagnosis of late-onset Cobalamin C is suspected and measurement of methylmalonic acid and total plasma homocysteine is indicated in case of neuropsychiatric symptoms and signs. The peculiarity of this patient is that until then she had not shown neuropsychiatric illness and the plasmatic homocysteine was only measured because of the IUFD. This allowed correct diagnosis of the metabolic disease.

This case suggests that women with late-onset cblC defect can have a successful pregnancy provided that physicians treat this metabolic disease adequately. The pre-pregnancy counselling and metabolic evaluation is the mainstay of the management of metabolic diseases in childbearing women. It is also important to report both management and outcomes of pregnancy in order to improve our knowledge of rare metabolic diseases.

## Data Availability

All data are included in this published article.
